# To Our Readers

**Published:** 1876

**Authors:** 


### Editorial and Miscellaneous.

All communications, of every character, must be addressed to Dr. Thos. S. Powell, 86 Pryor street.

Send money by check, postal order, or registered letter. We are willing to endorse for the honesty of our subscribers, but cannot be responsible for irregularity of the mails, or forgetfulness of friends. Address all to Dr. T. S. Powell.

Write your name, post-office, county and State plainly. Be sure to say to which post-office you wish The Record sent, and always date your letters and head them with your own post-office.

We receive many well-written letters, but doubt the authors being able to decipher their signatures. Communications for publication must be plainly and carefully written, on only one side of the paper. Don't forget this.

*To Our Readers*—The senior editor and business manager is authorized to say that The Record is published in the interest of the profession, and not for the pecuniary benefit of the editors, hence we desire every reader to feel an individual interest in its welfare, a personal interest in its success and continuous improvement. Will you not by your influence and personal endeavor, induce at least one physician of your acquaintance to subscribe for the Record, and commence with this number? You can give any reasonable time to remit the subscription fee. Please see or write to one friend before this number is exhausted.

